# Safety and Feasibility of Transperineal Targeted Microwave Ablation for Low- to Intermediate-risk Prostate Cancer

**DOI:** 10.1016/j.euros.2022.10.004

**Published:** 2022-10-22

**Authors:** Marco Oderda, Alessandro Marquis, Giorgio Calleris, Daniele D'Agate, Riccardo Faletti, Marco Gatti, Giancarlo Marra, Paolo Gontero

**Affiliations:** a Division of Urology, Department of Surgical Sciences, Molinette Hospital, University of Turin, Turin, Italy; b Division of Radiology, Molinette Hospital, University of Turin, Turin, Italy

**Keywords:** Focal therapy, Prostate cancer, Microwave, Koelis, Image fusion

## Abstract

**Background:**

Focal therapy has emerged as an interesting option for localized low- to intermediate-risk prostate cancer (PCa). Targeted microwave ablation (TMA) is a novel FT modality involving targeted delivery of microwave energy under multiparametric magnetic resonance imaging (MRI)/ultrasound guidance.

**Objective:**

To describe the step-by-step procedure for TMA and report early functional outcomes.

**Design, setting, and participants:**

This was an experimental phase 1–2 trial in 11 patients diagnosed with a single, MRI-visible PCa lesion of up to 12 mm, scored as International Society of Urological Pathology grade group (GG) 1 or 2.

**Surgical procedure:**

Transperineal TMA under MRI/ultrasound image fusion guidance.

**Measurements:**

We recorded patient and PCa features; intraoperative and postoperative parameters; pain (Visual Analog Scale [VAS]) and adverse events (Common Terminology Criteria for Adverse Events v5.0); and prostate-specific antigen (PSA), International Prostate Symptom Score (IPSS) and International Index of Erectile Function (IIEF-5) scores at 1 wk and 1, 3, and 6 mo.

**Results and limitations:**

The median patient age was 67 yr (interquartile range [IQR] 18). Median PSA was 5.4 ng/ml (IQR 1.8), median prostate volume was 51 cm^3^ (IQR 35), and median lesion size on MRI was 10 mm (IQR 4). Ten patients had GG 2 PCa and one had GG 1 disease. The median procedure time was 40 min (IQR 30). No intraoperative complications were reported. All treatments were performed on a day-case basis and no patients were discharged with a urinary catheter. Postoperatively, no grade ≥2 complications were reported. No significant changes in PSA (*p* = 0.46), IPSS (*p* = 0.39), or IIEF-5 scores (*p* = 0.18) scores were reported. The postoperative VAS score at 24 h was 0 for all patients.

**Conclusions:**

TMA is safe, feasible, and well tolerated in patients with low- to intermediate-risk PCa. Oncological outcomes are still awaited.

**Patient summary:**

Targeted microwave therapy is safe and feasible for selected patients with low- to intermediate-risk prostate cancer. The procedure is well tolerated and does not require a urinary catheter after the procedure. Cancer control outcomes are still awaited.

## Take Home Message

Target microwave ablation (TMA) is a novel focal therapy modality for low- to intermediate-risk prostate cancer involving targeted delivery of microwave energy under magnetic resonance imaging/ultrasound fusion guidance. TMA is performed in a day-case setting with good tolerability and no serious adverse events.

## 1 Introduction

In recent years, focal therapy (FT) has emerged as an interesting option for localized low- to intermediate-risk prostate cancer (PCa) with the aim of offering a personalized and less invasive treatment in comparison to radical therapies, reducing functional morbidity while maintaining oncologic efficiency [1]. The principle underlying FT is treatment of the “index lesion”, the substantial lesion that predicts PCa outcomes and from which other cancer clones can arise [2]. For successful FT, accurate detection of PCa foci via multiparametric magnetic resonance imaging (mpMRI) is essential, although systematic biopsies ensure the most accurate sampling possible [3]. Examples of established FT technologies are high-intensity focused ultrasound (HIFU), cryotherapy, and, more recently, focal laser ablation, vascular targeted photodynamic therapy, and irreversible electroporation. Depending on the FT type, the template ranges from focal ablation up to a “hockey-stick” ablation. In general, all FT modalities share a favorable toxicity profile, with most complications classified as mild [4]. Acceptable medium-term cancer control has been reported for men with intermediate-risk disease undergoing focal HIFU or cryotherapy [5].

Targeted microwave ablation (TMA) is a novel FT modality involving targeted delivery of microwave energy under mpMRI/ultrasound guidance using a Koelis Trinity (Koelis, Meylan, France) fusion system. Microwave propagation depends mainly on the permittivity of the medium rather than thermal conductivity or tissue impedance, and causes coagulative necrosis in a more predictable and controllable way in comparison to other energy sources [6].

## 2 Patients and methods

We performed a single-center, prospective, interventional phase 1–2 trial to evaluate the outcomes of TMA administered to 11 patients with newly diagnosed low- to intermediate-risk PCa. The study was in accordance with the Declaration of Helsinki and was approved by the local ethics committee (reference 0005732). It was registered on ClinicalTrials.gov as NCT04627896. The inclusion criteria were: a single MRI-visible lesion ≤12 mm diagnosed as International Society of Urological Pathology grade group (GG) ≤2 at biopsy; prostate-specific antigen (PSA) <20 ng/ml, a safety distance of 5 mm from the apex and rectum; and no signs of capsular involvement. No PCa was found outside the MRI targets. No limitations in terms of prostate volume were considered. All patients were diagnosed via transperineal targeted and systematic biopsies performed using a Koelis Trinity system. Patients underwent TMA in the lithotomy position under sedation or spinal anesthesia. Antibiotic prophylaxis consisted of cefixime 400 mg/d for 3 d.

The step-by-step procedure is detailed in the accompanying video. A Koelis Trinity system was used to retrieve the three-dimensional (3D) model of the prostate generated during diagnostic biopsy from mpMRI/ultrasound image fusion. A Trinity 3D ultrasound probe was inserted transrectally and held with a Koelis Steady Pro mechanical arm. New image fusion was performed to obtain an updated 3D model of the prostate. The prostate contours were delineated by the operator, who performed virtual simulations of the elliptical treatment area according to the needle position and microwave settings. Once the virtual simulation was satisfactory, the operator inserted a 17G treatment needle transperineally into the prostate via a dedicated grid. A new fusion image was generated to check the position of the needle, and then microwave energy was delivered to the target area using a very low-loss microwave ablation system (TATO3; Biomedical Srl, Firenze, Italy). The microwave power was set to 12 W on the basis of a preclinical predictive ablation chart [7], [8], while the duration was decided according to the area to be treated.

Follow-up consisted of clinical visits and PSA measurement and International Prostate Symptom Score (IPSS) and International Index of Erectile Function (IIEF-5) questionnaires at 1 wk and 1, 3, and 6 mo. MRI was scheduled at 5 mo and rebiopsy at 6 mo. The primary endpoints were the safety of the treatment, defined as the absence of complications, and the efficacy, defined as the absence of tumor in the treated area at 6 mo. Pain was measured using the Visual Analog Scale (VAS) and adverse events were classified according to the Common Terminology Criteria for Adverse Events version 5.0 [9].

## 3 Results

**Fig. 1:**
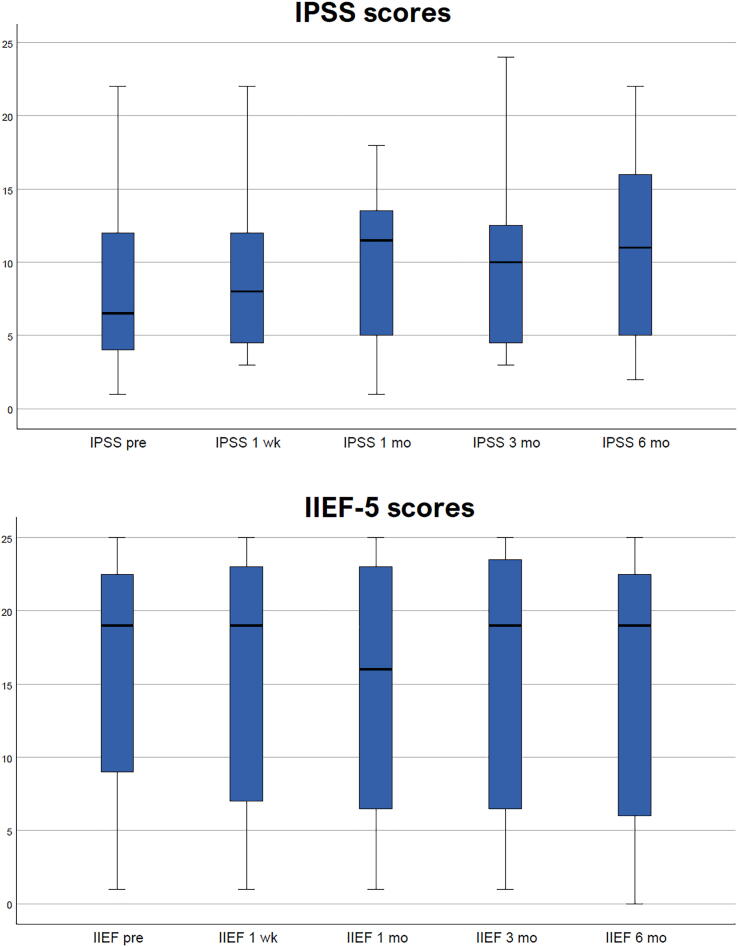
International Prostate Symptom Score (IPSS) and International Index of Erectile Function-5 (IIEF-5) results.

Eleven patients were recruited from September 2020 to March 2022. The median patient age was 67 yr (interquartile range [IQR] 18). Median PSA was 5.4 ng/ml (IQR 1.8), median prostate volume was 51 cm^3^ (IQR 35), and the median lesion Size on MRI was 10 mm (IQR 4). Ten patients were diagnosed with GG 2 Pca and one with GG 1 disease. The median procedure time was 40 min (IQR 30) and the median ablation time was 6 min (IQR 3), with nine patients needing two TMA sessions to adequately treat the cancer area. Periprocedural and postprocedural outcomes are shown in Table 1. No intraoperative complications were reported. All treatments were performed on a day-case basis and no patients were discharged with a urinary catheter. There were no cases of acute urinary retention. Postoperatively, no grade ≥2 complications were observed (Table 2). PSA levels did not significantly vary from baseline across the time points (*p* = 0.46). No significant changes in IPSS (*p* = 0.39) or IIEF-5 (*p* = 0.18) scores were reported, as shown in Figure 1. The postoperative VAS score at 24 h was 0 for all patients.

**Table 1:** Periprocedural and postprocedural outcomes

Parameter	Result
Anesthesia type, *n* (%)	
Spinal	10 (91)
Sedation	1 (9)
Median procedure duration, min (interquartile range)	40 (30)
Location of cancer, *n* (%)	
Posterior, basal	0 (0)
Posterior, equatorial	6 (54)
Posterior, apical	2 (18)
Transitional	1 (9)
Anterior, basal	0 (0)
Anterior, equatorial	2 (18)
Anterior, apical	0 (0)
Number of ablation sessions per patient, *n* (%)	
1	2 (18)
2	9 (82)
Energy used, *n* (%)	
12 W, 1 min	2 (10)
12 W, 2 min	4 (20)
12 W, 3 min	6 (30)
12 W, 4 min	7 (35)
12 W, 5 min	1 (5)
Median hospital stay, d (interquartile range)	1 (0)
Median postprocedural Visual Analog Scale pain score (interquartile range)	0 (0)
Intraoperative complications, *n* (%)	0 (0)

**Table 2:** Postoperative complications

Grade	Patients affected, *n* (%)				
	1 wk	1 mo	3 mo	6 mo	Overall
1 (mild)					
Hematuria	1 (9)	1 (9)	–	–	1 (9)
Urgency	1 (9)	–	–	2 (9)	2 (18)
Mild erectile dysfunction	1 (9)	–	–	–	1 (9)
2 (moderate)	–	–	–	–	0
3 (severe)	–	–	–	–	0
4 (life-threatening)	–	–	–	–	0
5 (death related to adverse event)	–	–	–	–	0

Our study can be regarded as phase 2a (development) according to the IDEAL recommendations on surgical innovations [10]. As a safety precaution, we chose strict inclusion criteria such as a small lesion size up to 12 mm. One prior feasibility study on transrectal TMA showed that post-treatment necrosis on MRI corresponded well to the predicted ablative zones, with a median largest necrosis dimension of 17.5 mm; the authors found total necrosis of the index tumor on MRI in eight out of ten patients [7]. Another safety measure was a distance of ≥5 mm from the apex and rectum to minimize the risk of complications. his goal was achieved, with no grade ≥2 complications observed, in line with the two preliminary studies published so far [7], [11]. The decision to use one or two energy delivery cycles was based on the shape of the MRI target and the prostate morphology to guarantee adequate treatment while remaining confined to the prostate to avoid potential complications. With transperineal access, we are able to angulate the probe in the sagittal plane in order to reach a satisfactory needle position more easily and avoid interference from the pubic arch for anterior lesions to a certain extent. However, it is foreseeable that it may not be possible to reach anterior areas for very large prostates, as happens for brachytherapy. In our study, the only two patients with anterior lesions had a prostate volume of 53 and 19 cm^3^. The issue of prostate size limits should be evaluated in further studies.

The median operative time was just 40 min, which is shorter than for most FT modalities. We performed almost all procedures under spinal anesthesia, but we showed the feasibility of TMA under sedation. Thanks to the precise organ-based tracking, the urethra was easily spared and there was no need for a urinary catheter postoperatively, minimizing discomfort for patients.

## 4 Conclusions

In conclusion, our study shows the safety, feasibility, and good tolerability of TMA for selected patients with PCa. Oncological outcomes are still awaited.

  ***Author contributions***: Marco Oderda had full access to all the data in the study and takes responsibility for the integrity of the data and the accuracy of the data analysis.

*Study concept and design*: Oderda.

*Acquisition of data*: Marquis, D’Agate, Gatti.

*Analysis and interpretation of data*: Oderda, Marquis.

*Drafting of the manuscript*: Oderda.

*Critical revision of the manuscript for important intellectual content*: Calleris, Faletti, Marra.

*Statistical analysis*: Oderda.

*Obtaining funding*: None.

*Administrative, technical, or material support*: None.

*Supervision*: Gontero.

*Other*: None.

  ***Financial disclosures:*** Marco Oderda certifies that all conflicts of interest, including specific financial interests and relationships and affiliations relevant to the subject matter or materials discussed in the manuscript (eg, employment/affiliation, grants or funding, consultancies, honoraria, stock ownership or options, expert testimony, royalties, or patents filed, received, or pending), are the following: Marco Oderda has received a speaker honorarium from Koelis. The remaining authors have nothing to disclose.

  ***Funding/Support and role of the sponsor*:** Koelis supported the study by providing the device and the disposables. The sponsor played no direct role in the study.

  ***Acknowledgments*:** We thank Fondazione Ricerca Molinette for their support of this study.
